# *C. elegans* outside the Petri dish

**DOI:** 10.7554/eLife.05849

**Published:** 2015-03-30

**Authors:** Lise Frézal, Marie-Anne Félix

**Affiliations:** Institute of Biology of Ecole Normale Supérieure, Centre National de la Recherche Scientifique, Paris, France; Institute of Biology of Ecole Normale Supérieure, Centre National de la Recherche Scientifique, Paris, France

**Keywords:** the natural history of model organisms, evolution, natural history, *C. elegans*

## Abstract

The roundworm *Caenorhabditis elegans* has risen to the status of a top model organism for biological research in the last fifty years. Among laboratory animals, this tiny nematode is one of the simplest and easiest organisms to handle. And its life outside the laboratory is beginning to be unveiled. Like other model organisms, *C. elegans* has a boom-and-bust lifestyle. It feasts on ephemeral bacterial blooms in decomposing fruits and stems. After resource depletion, its young larvae enter a migratory diapause stage, called the dauer. Organisms known to be associated with *C. elegans* include migration vectors (such as snails, slugs and isopods) and pathogens (such as microsporidia, fungi, bacteria and viruses). By deepening our understanding of the natural history of *C. elegans*, we establish a broader context and improved tools for studying its biology.

**DOI:** http://dx.doi.org/10.7554/eLife.05849.001

## Introduction

The free-living nematode *Caenorhabditis elegans* is a major model species that is used in a range of biological research. After initial work by Emile Maupas (Maupas, 1900) and Victor Nigon (Nigon, 1949; Nigon et al., 1960) on its mode of reproduction, meiosis and development, experiments by Sydney Brenner and collaborators in the 1960s and 1970s raised *C. elegans* to the status of a premier model organism. Besides its genome, the first to be sequenced for a multicellular organism (The C. elegans Sequencing Consortium, 1998), an extensive body of knowledge is now available on the molecular, cellular, developmental and behavioral biology (The C. elegans Research Community) of this organism. A number of key discoveries have been made by studying *C. elegans*, including the molecular mechanisms of apoptosis (Conradt and Xue, 2005) and gene silencing by small RNAs (Grishok, 2013).

The *C. elegans* reference strain N2 is a laboratory animal. This strain, originally isolated in Bristol, England, was cultured in the laboratory for many years before it was first frozen. Its laboratory environment consisted of agar plates seeded with *Escherichia coli* as a food source. *E. coli* was used because, as another model organism, it was already available in many laboratories—not because it was originally associated with wild *C. elegans*. An independent subculture from the same wild Bristol isolate was maintained in liquid axenic culture, and by comparing both strains, we now know of several mutations that appeared and were fixed in the N2 lineage, some of which appear adaptive in the agar plate environment. These mutations pleiotropically affect many traits, such as behavior, reproduction, susceptibility to pathogens, body size and entry into the dauer stage (where *C. elegans* undergo developmental arrest at the third larval stage) (McGrath et al., 2009, 2011; Duveau and Félix, 2012; Andersen et al., 2014; Green et al., 2014). Like other model organisms, *C. elegans* N2 has thus been modified by domestication.

For a century, the only information available on the natural history of *C. elegans* was that it could be found in compost heaps and in rich humus (Hodgkin and Doniach, 1997). From 2000 onward, this began to change when researchers embarked on an extensive sampling of wild *C. elegans* populations. Their efforts were motivated by wanting to develop *C. elegans* into a model organism for evolutionary biology and ecology research, which could also leverage the tools and knowledge already acquired for this species. In turn, exploring the natural history of *C. elegans* provides a context for, and also informs, basic biological research conducted with this organism, such as studies of its genome, development, behavior and immune system. In this article, we summarize our current knowledge of the natural history of this *C. elegans*, show how the isolation of natural pathogens of *C. elegans* informed basic biological research, and discuss a number of open questions.

## A rotting habitat and a boom-and-bust life cycle

**Figure 1.**
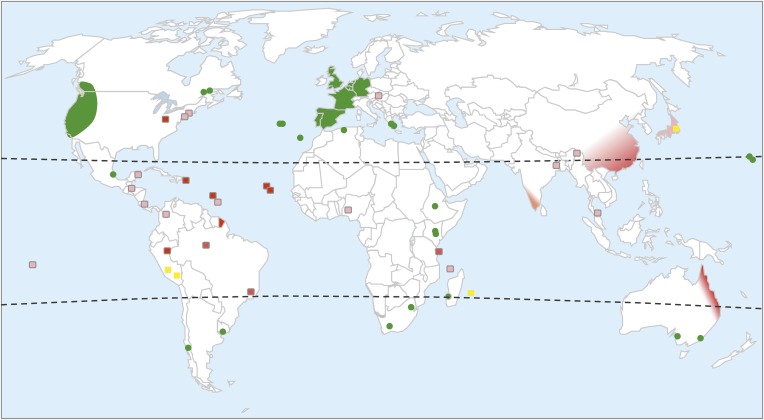
Worldwide distribution of *C. elegans*. Green shading highlights areas where *C. elegans* has been repeatedly collected. Green dots mark islands or locations where *C. elegans* has been collected at least once. Yellow squares represent areas where many Caenorhabditis species have been sampled and where *C. elegans* is present but rare (often found at altitude). Red shading highlights where *C. elegans* has never been collected despite the intensive sampling of many other *Caenorhabditis* species. Pink shading highlights where *C. elegans* has not been collected, despite the sampling of several other *Caenorhabditis* species. White represents areas that have never been sampled for *C. elegans* or very rarely. The distribution is inferred from published data (Abdul Kader and Côté, 1996; Barrière and Félix, 2005a, 2005b, 2007; Dolgin et al., 2008; Wang et al., 2010; Kiontke et al., 2011; Andersen et al., 2012; Félix and Duveau, 2012; Dey et al., 2013; Félix et al., 2013), WormBase, and our lab collection (http://www.justbio.com/worms/index.php). **DOI:** http://dx.doi.org/10.7554/eLife.05849.002

**Figure 2.**
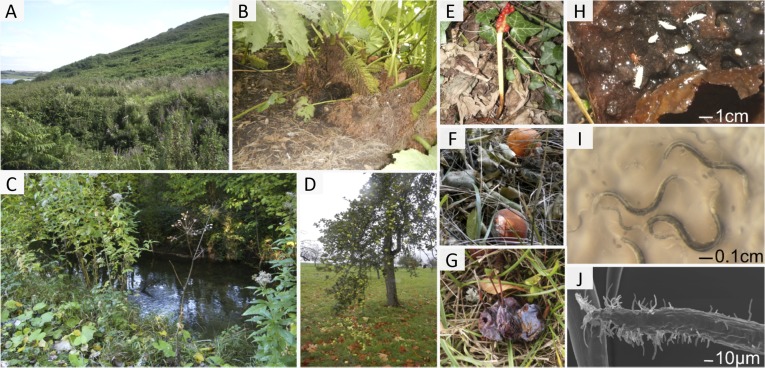
The habitat of *C. elegans* at different scales. (**A**–**D**) Landscapes that correspond to the macroscale *C. elegans* habitat; all are relatively humid areas where *C. elegans* has been found: (**A**) wet shrubland; (**B**) urban garden; (**C**) riverbank; and (**D**) fruit trees. (**E**–**G**) Bacteria-rich decomposing vegetal substrates, corresponding to the microscale *C. elegans* habitat: (**E**) *Arum* stem; (**F**) oranges and (**G**) plums. (**H**) Detail of a rotting apple at the stage where *C. elegans* proliferates. Springtails (white) and a mite are examples of animals that share the bacteria-rich habitat of *C. elegans* and that are potential carriers and/or predators (see also Table 1). (**I**) *C. elegans* nematodes on an *E. coli* lawn, just coming out of a rotten fruit. (**J**) Scanning electron micrograph of *C. elegans* infected with the fungus *Drechmeria coniospora*. Image credits: Marie-Anne Félix. **DOI:** http://dx.doi.org/10.7554/eLife.05849.003

*C. elegans* is found worldwide, predominantly in humid temperate areas (Figures 1 and 2A-D) (Kiontke et al., 2011; Andersen et al., 2012). This species was originally isolated in rich soil or compost (Hodgkin and Doniach, 1997), where it is mostly found in a non-feeding stage called the dauer (Barrière and Félix, 2005a, 2007). More recently, feeding and reproducing stages of *C. elegans* have been found in decomposing plant material, such as fruits and thick herbaceous stems (Figure 2E–G) (Félix and Duveau, 2012). These rotting substrates in their late stages of decomposition provide abundant bacterial food for the nematode. Like other model organisms, *C. elegans* is thus partially associated with human activity (cultivated fruits and stems, compost), but the species is also commonly found on stems and fruits in wilder settings, such as woods (Félix and Duveau, 2012). New types of habitat and geographical locations may still be discovered.

**Figure 3.**
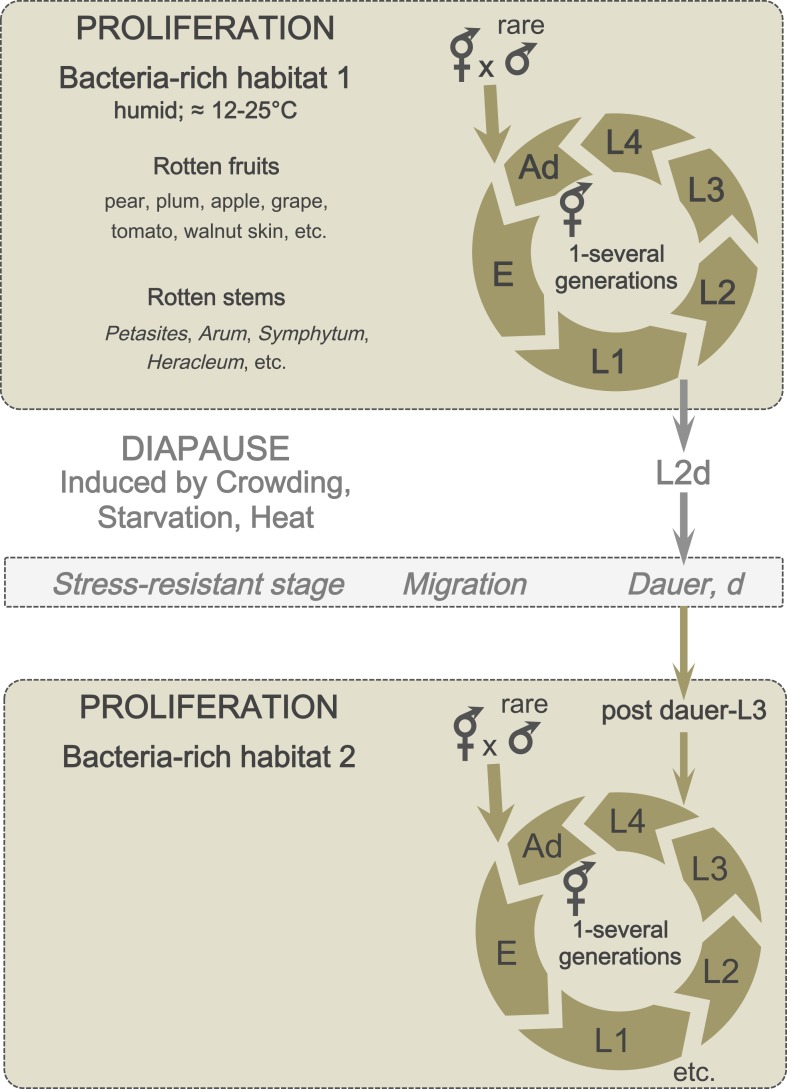
A schematic of *C. elegans* lifecycle in the wild. In bacteria-rich habitats (beige), the *C. elegans* life cycle begins with an embryonic (E) stage, followed by four larval (L1-L4) stages, and ends with an adult stage (Ad). Most animals are self-fertilizing hermaphrodites; males are rare and breeding with males therefore uncommon. Under suboptimal conditions (such as crowding and starvation), L1 larvae can enter a predauer stage (L2d) followed by the diapause stage (dauer). When better conditions arise, dauers develop into postdauer L3 larvae and re-enter the lifecycle at the L4 stage. **DOI:** http://dx.doi.org/10.7554/eLife.05849.004

Two alternative life cycles have been described in the laboratory for *C. elegans*, depending on environmental conditions. If well fed, newly hatched individuals pass through four larval stages (L1, L2, L3, L4) and reach the adult stage after 3 days. Under stressful conditions (such as crowding, limited food supply, and heat stress), individuals can shift during the L1 stage to an alternative developmental route and enter a predauer stage (L2d), followed by the non-feeding diapause stage called dauer (an alternative L3 stage) (Figure 3). Dauer larvae are resistant to various stresses and can survive for several months without food. Upon their return to more favorable conditions, dauer larvae feed again and resume development.

**Figure 4.**
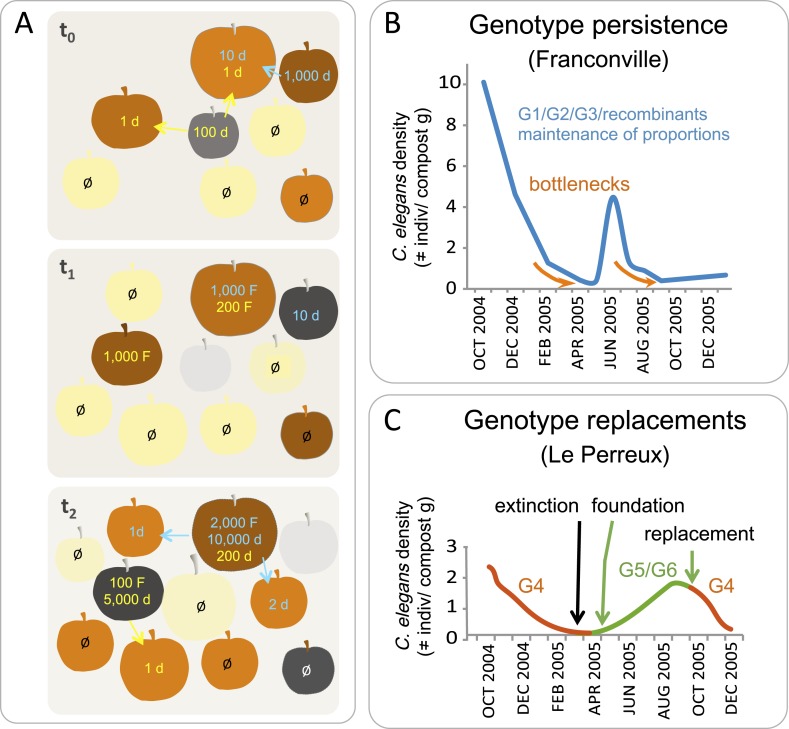
*C. elegans* population dynamics in a natural habitat. (**A**) A schematic of *C. elegans* population dynamics in an orchard. Population growth on a given apple has not been monitored to date and so is inferred here from data in (Félix and Duveau, 2012), based on many time points in an orchard and single time points on a given fruit. The fruits are shown at three time points (t) in identical positions on each panel, with t_0_ the first and t_2_ the last timepoint. Fruit colors indicate the degree of fruit decomposition, from early stages (yellow) to brown and dark grey (later stages), until disappearance (light grey). The number of feeding (F) and non-feeding dauer (d) individuals are indicated in color, with different colors representing different genotypes. Ø represents no colonization of a fruit by *C. elegans*. Arrows indicate dauer migration. How often different fruits or stems are colonized by several genotypes remains to be tested (Barrière and Félix, 2007; Andersen et al., 2012). (**B**–**C**) Actual population dynamics at the scale of a compost heap, from Barrière and Félix (2007). (**B**) In the first (Franconville) example, three main genotypes, G1, G2 and G3, persist in the heap at similar frequencies over the time period shown. In the second example (Le Perreux-sur-Marne), a single genotype, G4, was present, became extinct, then two new genotypes, G5 and G6, founded a new population. G4 reappeared in September, while G8 and G9 (not shown) disappeared. Genotypes were characterized using microsatellite markers. **DOI:** http://dx.doi.org/10.7554/eLife.05849.005

Population demographic surveys at the local scale in orchards and woods indicate that *C. elegans* has a boom-and-bust lifestyle (Félix and Duveau, 2012). *C. elegans* metapopulations evolve in a fluctuating environment where optimal habitats are randomly distributed in space and time (Figure 4A). A cycle of colonization of a food source likely begins when one to several dauer larvae discover a fruit or stem, exit the dauer stage and seed a growing population of up to 10^4^ feeding nematodes at different life-cycle stages (Figure 3). Some moderate-sized populations found in rotting fruits and stems do not contain any dauer larvae, but larger ones always include only adults, L1, L2d and dauers (Félix and Duveau, 2012). As a food source runs low, dauers may leave it to explore the neighboring environment for new islands of resources. Most of them will fail.

Developmental regulation and the behavior of dauer larvae are central to the *C. elegans* lifestyle. Dauer larvae display active locomotion and a specific behavior called nictation, where they stand on their tail and wave their body in the air. Remarkably, dauers may also congregate to form a column and nictate as a group (Félix and Duveau, 2012) (see the video; http://www.wormatlas.org/dauer/behavior/Images/DBehaviorVID4.mov). These behaviors are thought to help dauers to find passing invertebrate hosts that they can use for their dispersal, such as isopods, snails and slugs. Together, dauer physiology and behavior suggest that this developmental stage plays a key role in *C. elegans'* stress resistance, long-distance dispersal, and possibly its overwintering capacity.

Over the year, in surveys performed in France and Germany, *C. elegans* populations in rotting fruits typically peak in the fall, with proliferation possible in spring through to early winter (Félix and Duveau, 2012; Petersen et al., 2014). This seasonal dynamic is consistent with that observed in a semi-natural habitat, such as a compost heap, where *C. elegans* population size varies over the year, from a dense population in autumn to one that declines and undergoes bottlenecks in winter (Figure 4B) (Barrière and Félix, 2007; Petersen et al., 2014). These bottlenecks can lead to local extinctions. Where this happens, a new genotype may colonize the compost heap when favorable conditions return (Figure 4C).

The spatio-temporal distribution of *C. elegans* genetic diversity reflects this boom-and-bust lifecycle coupled to active migration. At the global scale, *C. elegans* genetic diversity is low and displays little geographical structure, which must be due to long-distance migration, perhaps aided by larger hosts, such as birds, rodents or humans (see Box 1). In contrast, at a local scale, founder effects have a huge impact, with low diversity in a given sample contrasting with high molecular diversity but weak haplotype diversity at the scale of a square kilometer (Barrière and Félix, 2005a; Haber et al., 2005; Sivasundar and Hey, 2005; Andersen et al., 2012).

10.7554/eLife.05849.006

Box 1. **Outstanding questions about the natural history of *C. elegans*.**

- What is the pattern of *C. elegan*s migration at different geographical distances? What are the relevant vectors? Are humans now major vectors over large scales?

Over a small scale, ongoing local population surveys will provide insights into migration patterns. Over large scales, selective sweeps estimated to date from 100–200 years ago have spread to different continents: their migration and selection might thus relate to human activity (e.g., via human travelling and the trafficking of goods, and via selection, perhaps by chemical compounds) (Andersen et al., 2012).

- What is *C. elegans* geographical center of origin? Are there species that are very closely related to *C. elegans* (see phylogeny in Box 2)?

Increased sampling in poorly sampled and isolated geographical regions might provide access to divergent populations that did not undergo the recent selective sweeps and so inform us of *C. elegans'* older history and habitats.

- What does *C. elegans* eat?

*C. elegans* likely feeds mostly on bacteria, but we do not know which bacteria. The sterols it requires might come from yeast or from the rotting plant substrate itself.

- What is the generation time and the number of generations per year in the wild?

The latter is surely far less than in the laboratory (ca. 150), but is it by one or two orders of magnitude?

- What are the relevant dauer entry and exit cues?

Whether associated organisms have an influence on dauer formation has not been investigated.

- Where does *C. elegans* spend its winter? What are the source populations?

In temperate regions, *C. elegans* is most often sampled in autumn, when it is found at high density. We ignore how and where this organism survives winter, for example, whether it becomes associated with a carrier organism.

**DOI:** http://dx.doi.org/10.7554/eLife.05849.006

## C. elegans sex life

*C. elegans* has a very peculiar mode of reproduction called androdioecy. It can reproduce either by self-fertilizing (selfing) hermaphrodites or by hermaphrodites (XX) breeding with males (X0). Hermaphrodites develop a female soma, and early in adulthood produce sperm in limited quantities (ca. 200–300 sperm cells). Males occur by non-disjunction of the X chromosomes at meiosis or in the progeny of male-hermaphrodite crosses. The non-disjunction of X chromosomes occurs overall at a low frequency (e.g., 0.1%) in the laboratory, and this frequency varies with genotype and the environment (Hodgkin and Doniach, 1997; Teotonio et al., 2006). In natural populations, outcrossing does occur but the overall proportion of males is low, generally similar to the estimated rate of chromosomal non-disjunction measured in the lab (Barrière and Félix, 2007; Cutter et al., 2009; Rockman and Kruglyak, 2009; Andersen et al., 2012; Félix and Duveau, 2012). In rare instances, outcrossing is evident from a high proportion of males or of heterozygotes in a given sample (Sivasundar and Hey, 2005; Barrière and Félix, 2007; Félix and Duveau, 2012).

This combination of metapopulation dynamics, a very low outcrossing rate and selection has a major impact on the *C. elegans* genome (Barrière and Félix, 2005b, 2007; Cutter, 2006; Cutter et al., 2009; Rockman and Kruglyak, 2009; Andersen et al., 2012). These three factors reduce the effective population size, and thus genetic diversity, and can wipe out diversity in a large genomic region. Indeed, positively selected alleles carry with them a large part of the genome by linkage, at the scale of megabases, particularly in the center of chromosomes where recombination is low. For example, most *C. elegans* isolates carry a recently swept genotype spanning a large part of chromosome V; several other chromosomes have also experienced detectable sweeps (Andersen et al., 2012). Because of high linkage disequilibrium due to selfing, selective sweeps may cover large fractions of chromosomes and have erased much genetic diversity. Background selection (the loss of non-deleterious variants due to selection against linked deleterious alleles) may further reduce linked genetic diversity in this selfing species (Rockman et al., 2010). As a consequence, genome-wide pairwise genetic diversity is low (ca. 10^−3^/bp), especially compared to outcrossing relatives (Jovelin et al., 2003; Cutter et al., 2009; Wang et al., 2010; Dey et al., 2013; Gimond et al., 2013). Overall, this means that much of the *C. elegans* genome has been shaped by selection at linked sites, not directly by selection at the locus of interest. These extreme sweeps also mean that recent events in historical times, perhaps mediated by humans, may have had a significant impact on *C. elegans*.

The selfing lifestyle of *C. elegans* also has an impact on phenomena such as inbreeding and outbreeding depression. Most animal species that reproduce by outcrossing suffer from inbreeding depression through the accumulation of deleterious recessive alleles. In a predominantly self-fertilizing organism, such as *C. elegans*, alleles are most often in a homozygous state due to repeated selfing, which allows deleterious recessive alleles to be purged by natural selection. Instead of inbreeding depression, wild *C. elegans* suffer from outbreeding depression (Dolgin et al., 2007). This phenomenon is best explained by the fact that selfing creates and maintains beneficial gene combinations, while outcrossing favors their disruption. Thus, the progeny of outcrossing are less fit than are the progeny of selfing. However, at least under certain circumstances of experimental evolution, outbreeding may contribute positively to *C. elegans* adaptation and inbreeding depression may reappear (Morran et al., 2009; Teotonio et al., 2012; Carvalho et al., 2014; Chelo et al., 2014).

Contributing also to a reduction in effective outcrossing, *C. elegans* possesses a widespread genetic incompatibility system, whereby a sperm poison (PEEL-1) arrests the development of embryos that do not synthesize the antidote (ZEEL-1) (Seidel et al., 2008, 2011). The *zeel-1* and *peel-1* genes are closely linked on chromosome I. The two allelic combinations, with both genes or without both genes, co-exist in the same geographical location, and the rest of the genome shows evidence of mixing. Yet this system, and other incompatibilities, may further contribute to lowering the effective outcrossing rate.

*C. elegans* has evolved selfing from an ancestor that reproduced through females and males (see Box 2). Male-specific genes are overall conserved yet appear to be subject to relaxed selection (Cutter, 2008; Thomas et al., 2012). Although *C. elegans* males still sense female pheromones from closely related species, hermaphrodites no longer produce pheromones to attract males (Chasnov and Chow, 2002; Chasnov et al., 2007), suggesting a partial degeneration of hermaphrodite outcrossing. On the male side, some aspects of mating behavior also appear to have degenerated, such as the ability to deposit a copulatory plug (Palopoli et al., 2008). The effect, and the future, of males in *C. elegans* natural populations remains an intriguing question.

10.7554/eLife.05849.007

Box 2. **The discovery and diversity of new *Caenorhabditis* species.**

Within the last 5 years, many *Caenorhabditis* species have been newly discovered, due to the collaborative, worldwide sampling of rotting fruits, flowers and stems for new *C. elegans* isolates and *Caenorhabditis* species (Kiontke et al., 2011). Today, 27 species are available in culture and many more are being isolated.

The genus is presently composed of two basal species, *C. plicata* and *C*. sp. 1, and two species supergroups, named *Elegans* and *Drosophilae*, which are further divided into species groups (Kiontke et al., 2011; Sudhaus, 2011; Félix et al., 2014; Huang et al., 2014) (see accompanying Box figure). The genus is globally distributed, with a higher species diversity in the tropics. *C. briggsae* is the only species that is abundant in both tropical and temperate climates. The three selfing species (shown in red on the accompanying figure) are spread over several continents, while some male-female species have regional distributions.

Most species were collected in microhabitats similar to those described for *C. elegans*. However, given the unavoidable bias in sampling, some species may have unexplored preferred microhabitats and animal vectors (Kiontke and Sudhaus, 2006). For example, *Caenorhabditis japonica* appears to be specifically associated with the hemipteran bug *Parastrachia japonensis,* which feeds in summer on fruits of *Schoepfia jasminodora* (Kiontke et al., 2002; Okumura et al., 2013a, 2013b).

These new findings enable comparative studies across the *Caenorhabditis* genus. For instance, closely related species, such as *C. briggsae* and *C. nigoni* (Woodruff et al., 2010), or *C. remanei* and *C. latens* (Dey et al., 2012), produce viable hybrids, thus rendering possible the dissection of mechanisms involved in speciation and of the transition from females to hermaphrodites. Phenotypic diversity in the genus also includes morphology, mating behavior (Kiontke et al., 2011), development and cell biology (e.g., Wang and Chamberlin, 2002; Félix, 2007; Baldi et al., 2009; Pénigault Félix., 2011; Riche et al., 2013; Barrière and Ruvinsky, 2014; Verster et al., 2014). An improved understanding of the ecology and the biology of the *Caenorhabditis* species, combined with comparative genomics, promise great insights into the evolutionary biology of the *Caenorhabditis* clade.

**Box 2—Figure 1.**
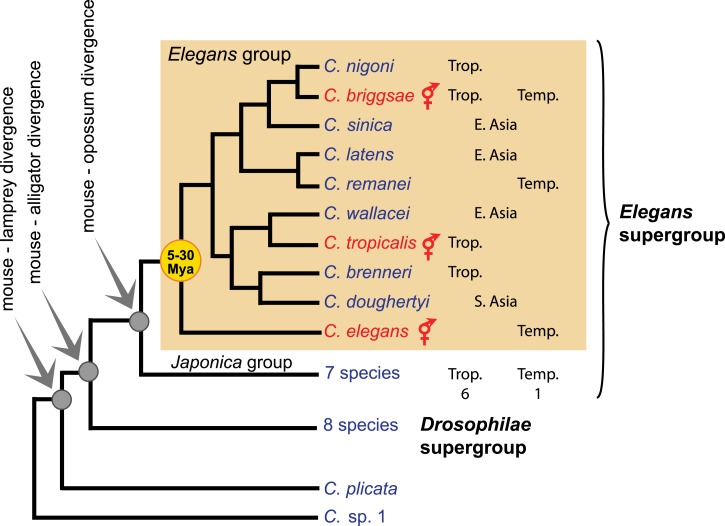
Phylogeny of the *Caenorhabditis* genus, with emphasis on the *Elegans* group, based on (Kiontke et al., 2011). Trop.: tropical distribution. Temp.: temperate. See http://worldwideworm.banshy.fr/ for geographical distributions. **DOI:** http://dx.doi.org/10.7554/eLife.05849.009

**DOI:** http://dx.doi.org/10.7554/eLife.05849.007

## Position in the food chain

*C. elegans* shares its microhabitat with arthropods and with other microorganisms (bacteria and fungi) and invertebrates (Table 1), including other nematodes such as *Oscheius*, *Pristionchus*, *Panagrellus* and other *Caenorhabditis* species, such as *Caenorhabditis briggsae* (Kiontke et al., 2011; Félix and Duveau, 2012). If not with *E. coli*, it is noteworthy that *C. elegans* shares its rotting fruit habitat with two other top model organisms, *Drosophila melanogaster* and *Saccharomyces cerevisiae* (Fay and Benavides, 2005; Diezmann and Dietrich, 2009; Schacherer et al., 2009; Charron et al., 2014). A specific association is actually found between another *Caenorhabditis* species and another *Drosophila* species: this nematode species, *C. drosophilae*, feeds on rotting cactus in desert areas and its dauer juveniles use a local *Drosophila* species as a vector to move between cacti (Kiontke, 1997).

**Table 1.** The biotic environment of *C. elegans* **DOI:** http://dx.doi.org/10.7554/eLife.05849.008

		Groups of organisms	Examples	References
Parasites		Fungi	*Drechmeria coniosporia, Harposporium* sp.	Jansson et al., 1985; Couillault et al., 2004; Félix and Duveau, 2012
		Microsporidia	*Nematocida parisii*	Troemel et al., 2008
		ss(+) RNA virus	Orsay virus	Félix et al, 2011
		Bacteria	*Serratia marcescens*	Pradel et al., 2007*
			*Elizabethkingia* sp.	Félix and Duveau, 2012
			*Leucobacter* spp.	Hodgkin et al., 2013
	Food		Others	Félix and Duveau, 2012
		Fungi	Yeasts	Félix and Duveau, 2012
		Other unicellular eukaryotes?		Félix and Duveau, 2012
Other nematodes		Rhabditidae	*Caenorhabditis briggsae*	Félix and Duveau, 2012
			*Oscheius*, *Rhabditis, Mesorhabditis* spp.	Félix and Duveau, 2012; Barrière and Félix, 2014
		Panagrolaimidae	*Panagrolaimus* and *Panagrellus* spp.	Félix and Duveau, 2012; Barrière and Félix, 2014
		Aphelenchs	Fungi-eating	Félix and Duveau, 2012; Barrière and Félix, 2014
		Diplogastrids	*Pristionchus* spp.	Félix and Duveau, 2012; Barrière and Félix, 2014
	Predators	Fungi	Trapping fungi	Maguire et al., 2011*
		Arthropods	Collembola?	Lee and Widden, 1996; Félix and Duveau, 2012
Vectors			Mites such as *Sancassania* sp.	Félix and Braendle, 2010; Félix and Duveau, 2012
			Other arthropods	Sudhaus and Kühne, 1989; Sudhaus and Kiontke, 1996; Kiontke and Sudhaus, 2006
		Mollusks	Snails, slugs	Sudhaus and Kiontke, 1996; Barrière and Félix, 2005b; Caswell-Chen et al., 2005; Barrière and Félix, 2007; Félix and Duveau, 2012

Some organisms may act as both food and pathogen, or as both vector and predator. Other nematodes may be competitors for food but also predators. The predatory relationships are inferred from the co-occurrence of the two species in the wild, from laboratory predation assays, and, in the case of mites and fungi, by observation on the laboratory isolation plate; specific studies would be needed to observe them directly in a natural setting.

* and our unpublished observations.

The *C. elegans* diet mainly consists of bacteria and small eukaryotes (Félix and Braendle, 2010; Félix and Duveau, 2012), but has not been further characterized. The trophic interaction between the nematode and its prey is not univocal: as *C. elegans* adults grow old, they can serve as food to the same microorganisms.

Predators of *C. elegans* are also little studied. From co-occurrence in the wild and from laboratory experiments, possible natural predators include small arthropods, such as mites or springtails (Figure 2H), other nematodes, such as *Pristionchus* spp., and trapping nematophagous fungi (Lee and Widden, 1996; Kiontke and Sudhaus, 2006; Bento et al., 2010; Maguire et al., 2011; Félix and Duveau, 2012) (Table 1).

*C. elegans* also constantly interacts with a variety of obligate and non-obligate parasites, such as fungi, microsporidia, bacteria and viruses (Troemel et al., 2008; Félix et al., 2011; Félix and Duveau, 2012; Hodgkin et al., 2013) (Table 1). These parasites infect their host via the two most exposed parts of the nematode, the cuticle and the intestine. Some non-invasive bacteria form a biofilm along the nematode's cuticle or directly stick to it (Hodgkin et al., 2013). Other bacteria proliferate in the nematode gut, which may induce constipation and likely impairs nutrient uptake (Félix and Duveau, 2012). The most intrusive parasites enter and proliferate inside the nematode body. Some pierce the cuticle (e.g., *Drechmeria coniospora* [Couillault et al., 2004], Figure 2J), while others enter intestinal cells via the apical membrane (e.g., microsporidia and Orsay virus [Troemel et al., 2008; Félix et al., 2011]).

## A model organism strengthened by its natural history

Numerous characteristics of *C. elegans* biology arise from its natural history and explain why this animal has become such an emblematic species for scientists. While some of these characteristics were clear for the early pioneers of *C. elegans* research, others were only discovered later.

First, like other model organisms, *C. elegans* rapidly proliferates in the wild upon encountering a food source, and can remain in a resting stage for long periods of time. In the lab, these features translate into organisms with short generation times (for their group) and good storage ability. Indeed, *C. elegans* displays a short generation time compared with many other nematodes and can simply be maintained on cholesterol-supplemented agar plates and fed *E. coli*. The natural ability of dauer larvae to live for several months offers a convenient option for short-term storage. *C. elegans* survives freezing, which enables their long-term storage at −80°C or in liquid nitrogen—an extremely useful feature for genotype storage and experimental design.

Second, the natural mode of reproduction of *C. elegans* makes this nematode a dream come true for geneticists. Selfing permits the generation of homozygous stocks, without inbreeding depression. The ability to induce males makes genetic crosses easy.

Third, *C. elegans* egg shells are naturally resistant to many environmental stresses, allowing the use of bleach. Bleaching is widely employed in the laboratory: in a population, only embryos will survive bleaching, thus allowing synchronization and removal of microbial contaminants.

Finally, a convenient feature of the reference *C. elegans* strain, N2, that is found in some but not all *C. elegans* wild isolates or *Caenorhabditis* species is an active RNA interference pathway. This pathway was perhaps selected in response to encounters with viruses and transposons (Tijsterman et al., 2002; Félix, 2008; Félix et al., 2011).

## Recent findings informed by natural history

The exploration of the natural habitat of *C. elegans* opens new doors to study its biology, for example, its development, physiology and behavior. Strikingly, for a top model organism such as *C. elegans*, a large proportion of its genes are, as yet, not associated with any mutant phenotype. Our exploration of its natural history advances biological research in two key respects. First, the quest for wild isolates of *C. elegans* has yielded deletion mutants for many genes (Maydan et al., 2007; Thompson et al., 2013). Second, our improved knowledge of its wild habitat gives us new environmental parameters to test for mutant phenotypes.

As an example, an obvious ecological parameter is the interaction between *C. elegans* and its natural parasites, which have likely had a considerable impact on its recent evolution. From genomic analyses, expanded gene families, such as ubiquitin-adaptor genes, were hypothesized to have evolved under diversifying selection provided by pathogens or toxins (Thomas, 2006). Recently, a ubiquitin-mediated response was indeed shown to take part in the *C. elegans* defense against two wild parasites, *Nematocida parisii* (microsporidia) and the Orsay virus (Bakowski et al., 2014), as well as in behavioral avoidance of a bacterial pathogen (Chang et al., 2011).

The isolation of the Orsay virus, an RNA virus, was also instrumental in demonstrating in *C. elegans* the antiviral role of genes involved in RNA interference (so far studied in response to artificially administered double-stranded RNAs) (Félix et al., 2011). Moreover, an exploration of natural variation in *C. elegans* identified, by a genome-wide association study, a locus called *drh-1*. This locus was shown to carry in many *C. elegans* isolates a deletion that renders them permissive for Orsay virus replication. The *drh-1* gene is homologous to the *RIG-I* family of genes that encode mammalian viral sensors. In *C. elegans*, this likely upstream sensor of viral RNAs was shown to activate an antiviral small RNA pathway (Ashe et al., 2013). Further molecular immunity pathways will likely be unraveled by studying the interaction of *C. elegans* with its natural associates (Liu et al., 2014) and its corresponding natural phenotypic variation.

How *C. elegans* development and behavior are modulated by the environment will also be greatly enriched by a better knowledge of its natural history. A key component of its population cycles that remains to be explored is the regulation of dauer entry and exit by relevant environmental factors, besides crowding and high temperature. Behaviorally, the dauer larva has been little studied; for example, we still know little about its olfactory responses. Recent work has demonstrated the importance of a specific set of neurons (called IL2 cells) and of cholinergic transmission in controlling dauer nictation (Lee et al., 2011). When present at high density, the dauers can nictate in large groups (Félix and Duveau, 2012). The mechanistic basis and environmental regulation of this host-seeking behavior are still poorly understood.

Another striking resource provided by the exploration of *C. elegans* natural history is the phenotypic diversity at the intra- and inter-specific level (see Box 2). As an example, the mode of reproduction has been modified multiple times in the *Caenorhabditis* genus. While most species reproduce through males and females, *C. elegans*, *C. briggsae* and *Caenorhabditis tropicalis* are exceptions by having evolved spermatogenesis in a female soma. Phylogenetic studies and molecular dissection of germ line sex determination argue for a convergent evolution of androdioecy, three times in the *Caenorhabditis* genus (Guo et al., 2009; Kiontke et al., 2011; Thomas et al., 2012). Another opening provided by the huge rise in *Caenorhabditis* species and strain collections is the ability to study reproductive isolation and speciation mechanisms in this group (Woodruff et al., 2010; Kiontke et al., 2011; Dey et al., 2012).

## Conclusion

Despite intense laboratory work on *C. elegans*, many important features of this species in the wild remain mysterious (Box 1). We still know very little concerning this nematode's feeding, dauer entry and exit cues, migration, overwintering, and we have only just started to glimpse organisms associated with it, be they food or pathogens. A greater understanding of its natural history could greatly inform our studies of its physiology, behavior and immunity. Comparative studies between *Caenorhabditis* species also promise to provide a better understanding of the role and the evolutionary history of specific gene families and other orphan genes.

In addition, improving our understanding of *C. elegans'* natural history will help establish it as a model species for evolutionary and ecological studies. The diversity of modes of reproduction in the *Caenorhabditis* genus makes them attractive models for studies of the causes and consequences of such variation. Its boom-and-bust lifestyle is favorable for studies of *C. elegans* metapopulation dynamics in different environments.

To conclude, knowing more about *C. elegans'* life outside of the Petri dish is opening new avenues to laboratory research. As for other model organisms, it also helps us to realize how idiosyncratic some features of the *C. elegans* N2 reference strain are—only a snapshot in laboratory evolution.
